# Supplementary material for: Multi-biosensing hairband for emergency health assessment
Source: Nat Commun. 2025 Aug 5;16:7224. doi: 10.1038/s41467-025-62556-6 (PMC12325749; doi:10.1038/s41467-025-62556-6)
Supplement: Supplementary file 1 — Supplementary Information [file 41467_2025_62556_MOESM1_ESM.pdf]

Supplementary Information

Multi-Biosensing Hairband for Emergency Health Assessment

Ming Li[1], Ganghua Li[1], Tong Xu[1], Yiwen Wang[1], Ruidong Xu[1], Xinwei Zhang[1], Fuxing Chen[1], Ning Yu[2*], Mingwei Tian[1*]

[1] Research Center of Health and Protective Smart Textiles, State Key Laboratory of Bio-Fibers and Eco-Textiles, College of Textiles and Clothing, Qingdao University, Qingdao, 266071, P.R. China.

[2] Department of Anesthesiology, The Affiliated Hospital of Qingdao University, Qingdao, 266071, P.R. China.

[*] Corresponding author: yuning@qduhospital.cn*,mwtian@qdu.edu.cn*

Supplementary Fig. 1 Multi-scenario applications of the multi-biosensing hairband for emergency health assessment.

Supplementary Fig. 2 Schematic illustration of the weavable multi-biosensor array.

Supplementary Fig. 3 Contact angle of as-spun biosensor and textile substrate.

Supplementary Fig. 4 Wetting property of as-spun biosensor and textile substrate.

Supplementary Fig. 5 Schematic illustration of sweat directional transport to multi-biosensor array.

Supplementary Fig. 6 Fabrication of as-spun biosensors through wet spinning technique.

Supplementary Fig. 7 Schematic illustration and structural characterization of as-spun biosensors.

Supplementary Fig. 8 Electrochemical performance of CSCP yarn prepared with different CNT ratio.

Supplementary Fig. 9 Schematic illustration and structural characterization of Ag/AgCl yarn.

Supplementary Fig. 10 Stability of Ag/AgCl yarn in PBS solutions containing 5 mM and 10 mM of different saline solution.

Supplementary Fig. 11 Structural characterization of CSCP yarn.

Supplementary Fig. 12 Calibration curves of as-spun biosensor signals versus analyte concentration.

Supplementary Fig. 13 Reproducibility of as-spun biosensor signals versus analyte concentration.

Supplementary Fig. 14 Long-term stability of as-spun temperature sensor over 24 hours.

Supplementary Fig. 15 Selectivity of as-spun biosensors.

Supplementary Fig. 16 Long-term stability of as-spun biosensors within 60 days.

Supplementary Fig. 17 Sensitivity comparison of as-spun temperature sensor with other reported patch or fiber sensors.

Supplementary Fig. 18 Box-and-whisker plot of mouse epithelial cells cultured in biocompatibility test.

Supplementary Fig. 19 Stability of as-spun biosensors against environmental conditions.

Supplementary Fig. 20 Stability of as-spun biosensors under regular washing.

Supplementary Fig. 21 Schematic illustration and component list of the electronic system of circuit board.

Supplementary Fig. 22 On-body multimodal monitoring for health monitoring during exercise.

Supplementary Fig. 23 Distribution of physiological signals collected from five subjects during exercise.

Supplementary Fig. 24 Ex situ measurements of physiological signals collected from five subjects during exercise.

Supplementary Table 1 Comparison of the temperature sensing performance.

Supplementary Table 2 Comparison of the pH sensing performance.

Supplementary Table 3 Comparison of the Na^{+} sensing performance.

Supplementary Table 4 Comparison of the K^{+} sensing performance.

Supplementary Table 5 Comparison of the Ca^{2+} sensing performance.

Supplementary Table 6 Personal information of five volunteers participating in the on-

body sweat analysis using a multi-biosensing hairband.

Supplementary Video 1 (separate file) Continuous preparation of as-spun multi-biosensing yarns through the self-assembled coaxial wet-spinning device.

Supplementary Video 2 (separate file) Viscoelastic comparison of carboxylated carbon nanotube, silk fibroin, polylactic acid, and SCP mixture.

Supplementary Video 3 (separate file) On body application of the multi-biosensing hairband during exercise.

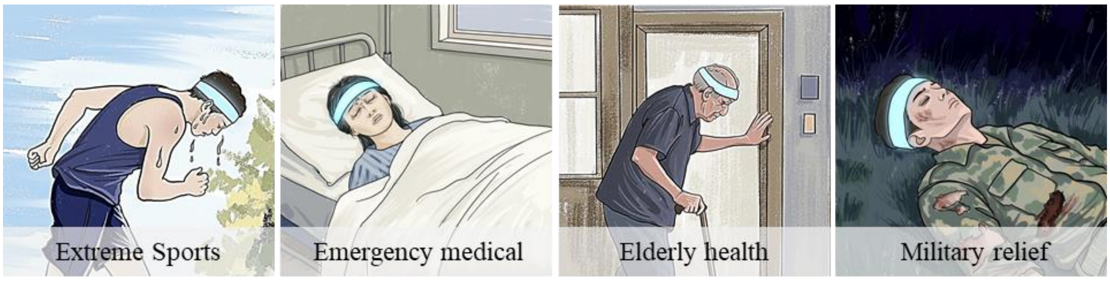

Supplementary Fig. 1 Multi-scenario applications of the multi-biosensing hairband for emergency health assessment.

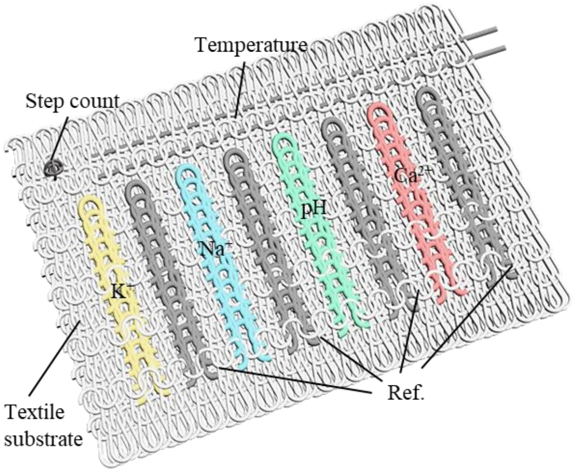

Supplementary Fig. 2 Schematic illustration of the weavable multi-biosensor array.

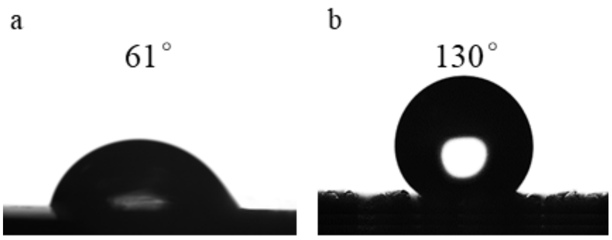

Supplementary Fig. 3 Contact angle of as-spun biosensor and textile substrate. a, b Optical image of a water droplet on as-spun biosensor (a) and hydrophobic treated textile substrate (b).

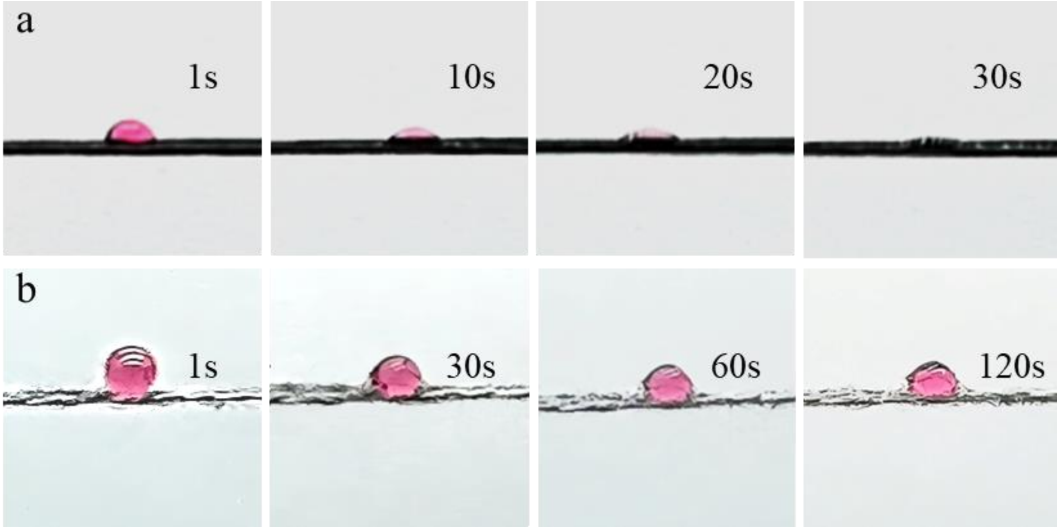

Supplementary Fig. 4 Wetting property of as-spun biosensor and textile substrate. a, b Diffusing process of the droplet on as-spun biosensor (a) and hydrophobic treated textile substrate (b).

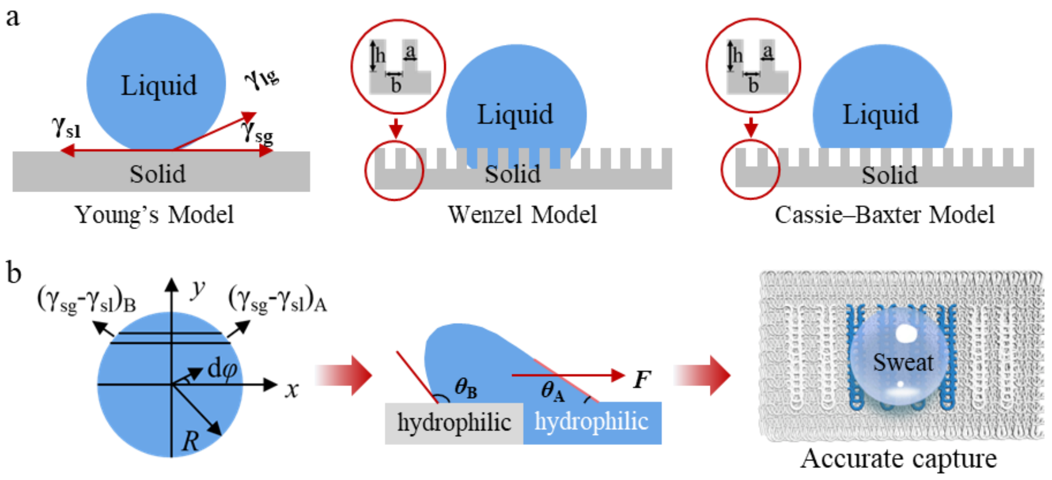

Supplementary Fig. 5 Schematic illustration of sweat directional transport to multi-biosensor array. a Wettability behavior of a liquid on the solid surface. b Sweat capture ability of multi-biosensor array.

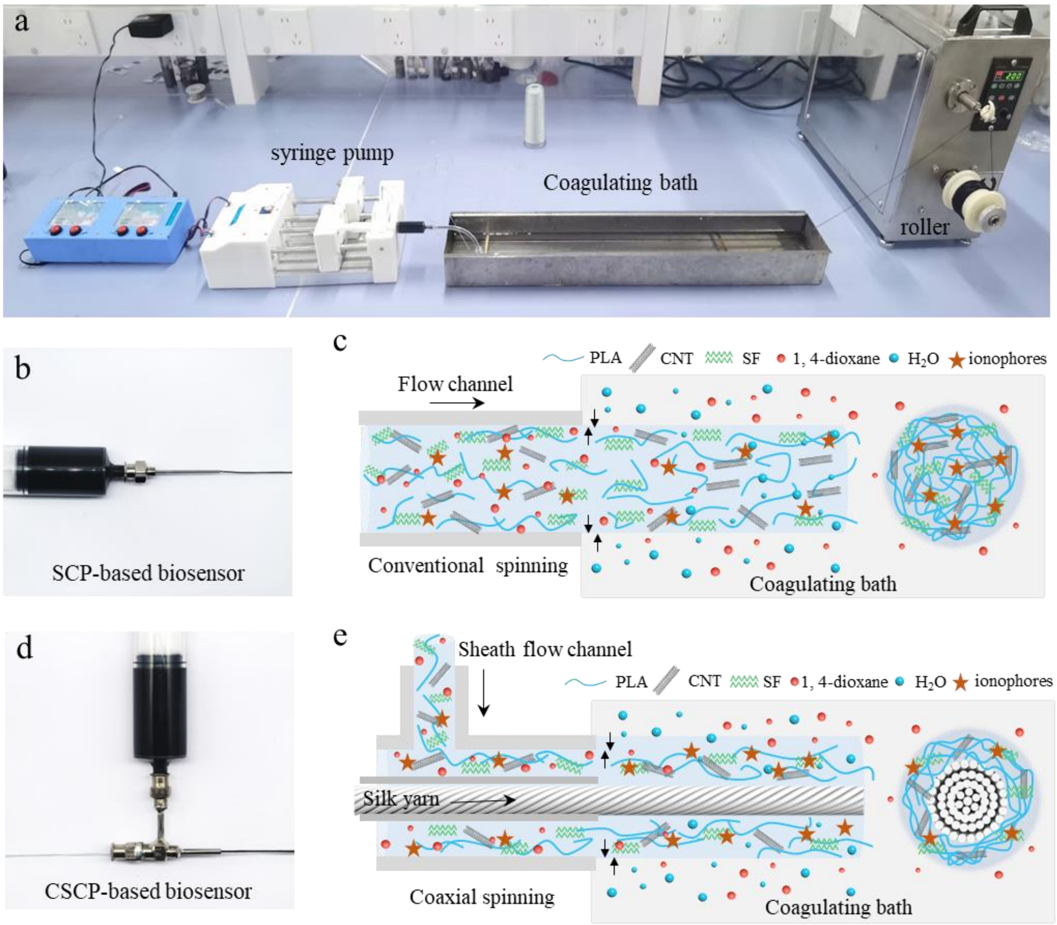

Supplementary Fig. 6 Fabrication of as-spun biosensors through wet spinning technique. a Continuous preparation of the biosensing yarn through self-assembled wet-spinning device. b, c Optical image and diagrammatic sketch of SCP-based biosensor preparation through conventional wet-spinning production. d, e Optical image and schematic diagrams of CSCP-based biosensor preparation through coaxial wet-spinning production.

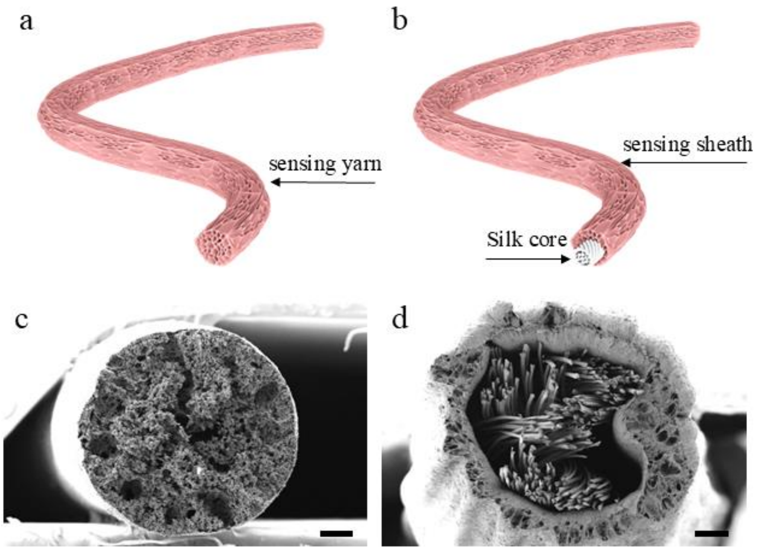

Supplementary Fig. 7 Schematic illustration and structural characterization of as-spun biosensors. a, b Schematic diagrams of SCP-based biosensor (a) and CSCP biosensor (b). c, d Cross-section SEM images of SCP-based biosensor (c) and CSCP biosensor (d). Scale bars, 100 μm.

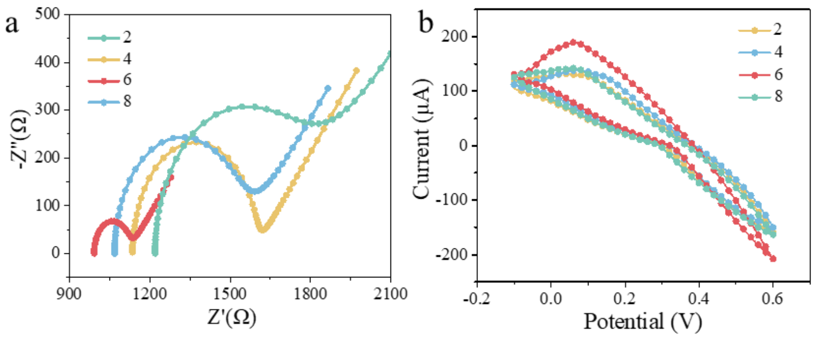

Supplementary Fig. 8 Electrochemical performance of CSCP yarn prepared with different CNT ratio. a, b Electrochemical impedance spectroscopy curves(a) and cyclic voltammetry curves(b) tested in 5 mM $K_3[Fe(CN)_6]$ containing 0.1 m KCl solution. Scan rates, 5 mV/s.

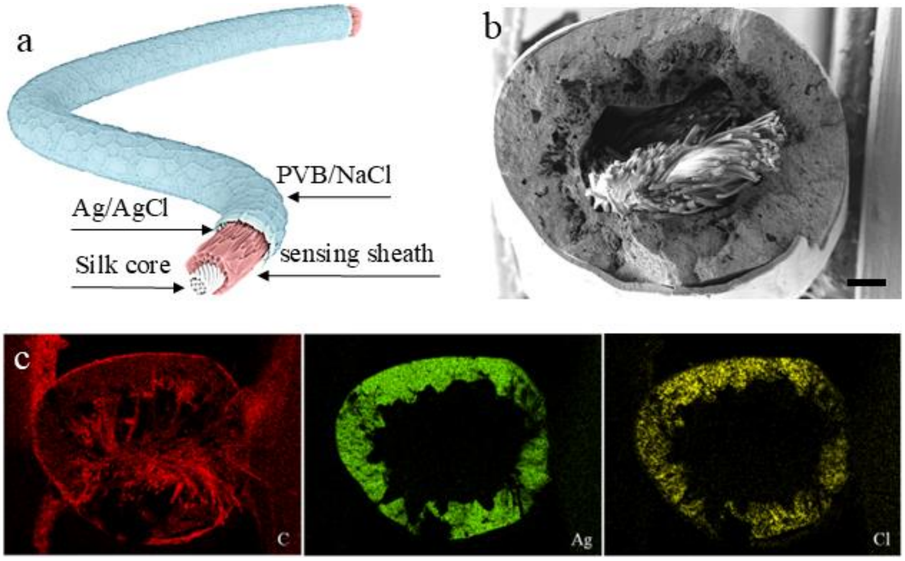

Supplementary Fig. 9 Schematic illustration and structural characterization of Ag/AgCl yarn. a Schematic diagram of Ag/AgCl yarn. b Cross-section SEM image of Ag/AgCl yarn. Scale bars, 100 μm. c EDS image of Ag/AgCl yarn.

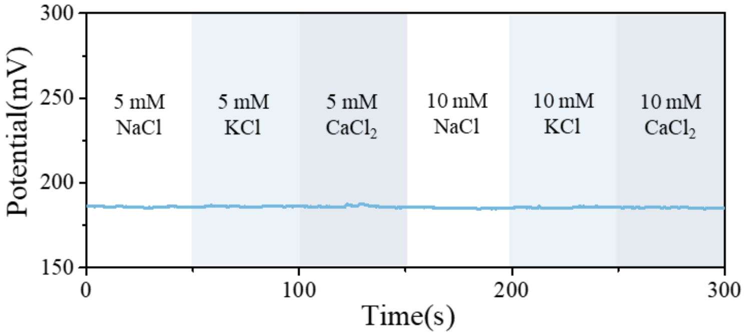

Supplementary Fig. 10 Stability of Ag/AgCl yarn in PBS solutions containing 5 mM and 10 mM of different saline solution.

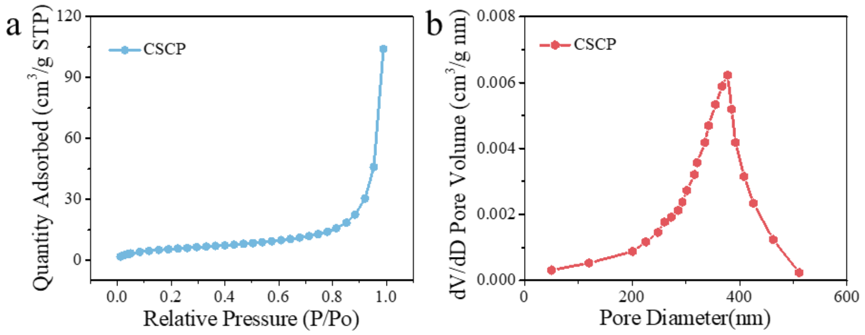

Supplementary Fig. 11 Structural characterization of CSCP yarn. a Adsorption–desorption isotherm of the as-spun multimodal sensors. Analysis adsorptive: N_2, Analysis bath temp.: -195.850 °C, Equilibration interval: 20 s. b Distribution curve of CSCP yarn (dV/dD—pore diameter curve).

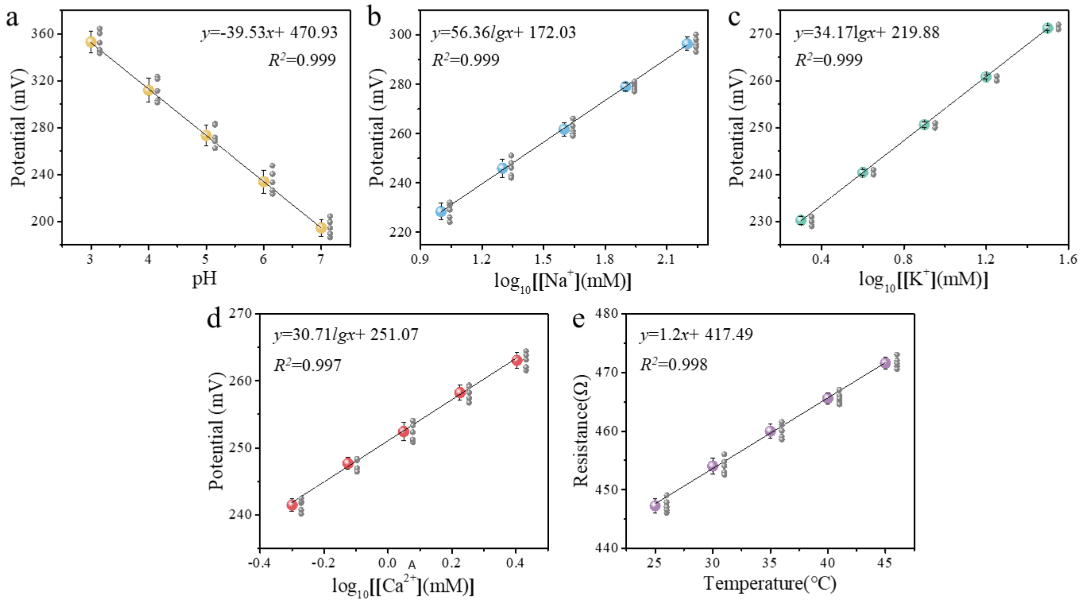

Supplementary Fig. 12 Calibration curves of as-spun biosensor signals versus analyte concentration. a-e Linear response of the (a) pH, (b) Na^+, (c) K^+, (d) $Ca^{2+}$and (e) temperature sensors (n = 5 independent biosensors). Data are presented as mean values +/− SEM.

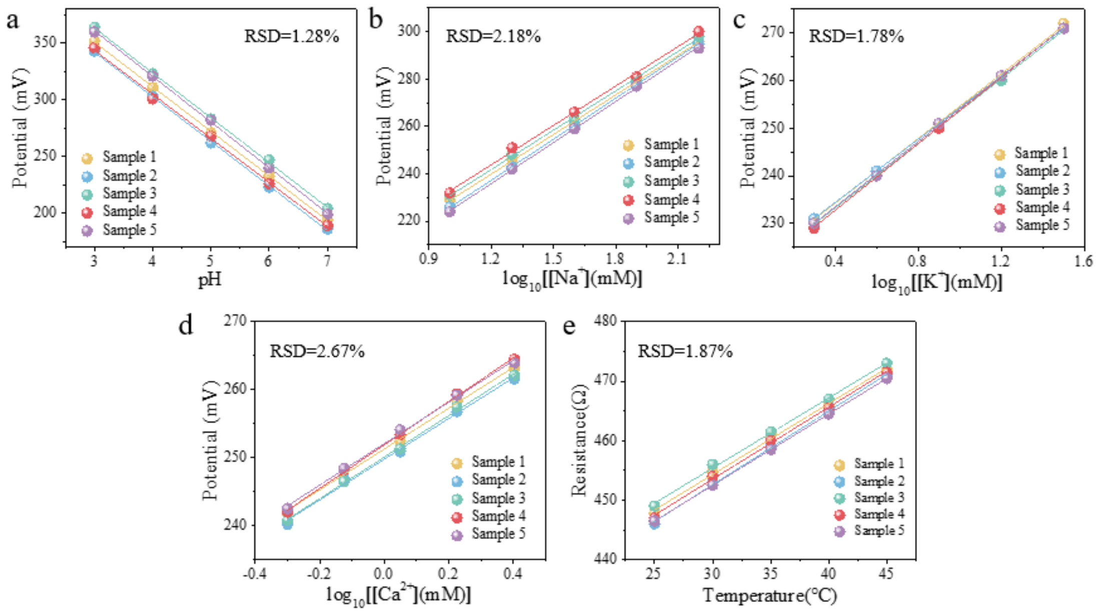

Supplementary Fig. 13 Reproducibility of as-spun biosensor signals versus analyte concentration. a-e Linear response of the (a) pH, (b) Na^{+}, (c) K^{+}, (d) $Ca^{2+}$and (e) temperature sensors.

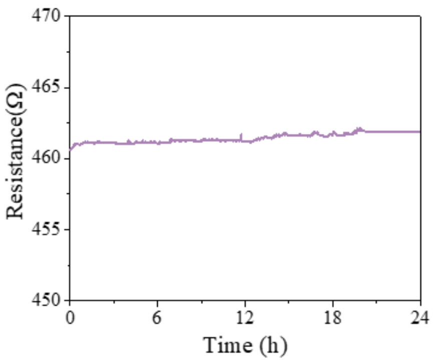

Supplementary Fig. 14 Long-term stability of as-spun temperature sensor over 24 hours.

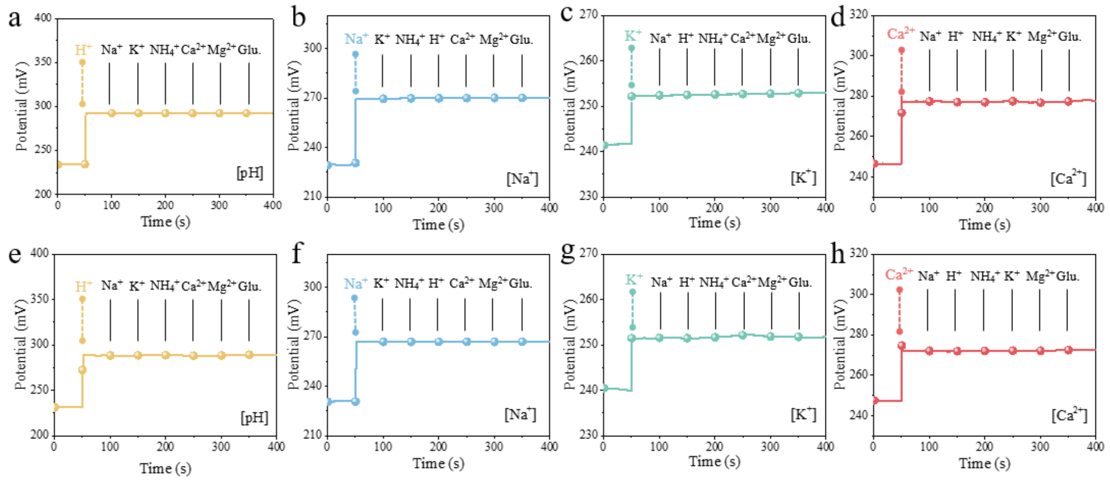

Supplementary Fig. 15 Selectivity of as-spun biosensors. a-h Selectivity with the interfering materials in 5 mM (a-d) and 10 mM (e-f).

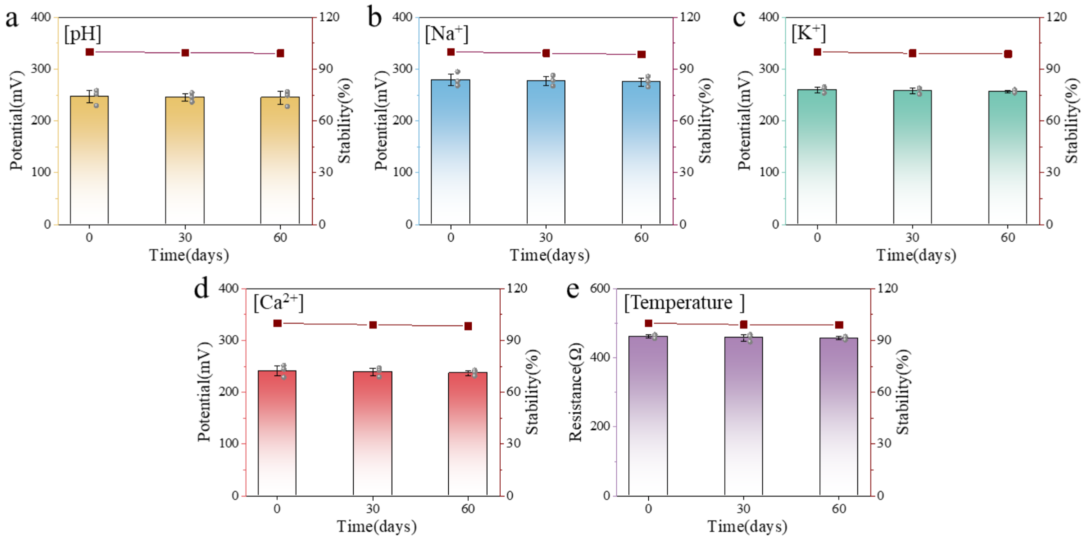

Supplementary Fig. 16 Long-term stability of as-spun biosensors within 60 days. a-e Change in sensitivity of the (a) pH, (b) Na^+, (c) K^+, (d) $Ca^{2+}$and (e) temperature sensors (n = 3 independent biosensors). Data are presented as mean values +/− SD.

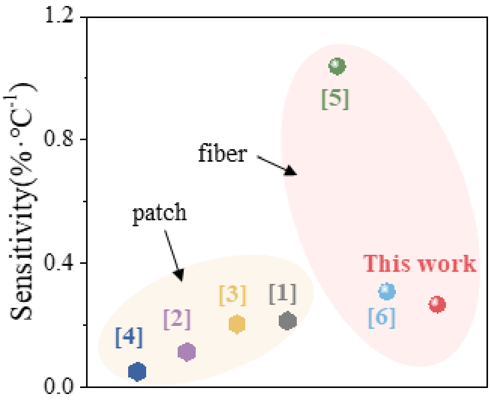

Supplementary Fig. 17 Sensitivity comparison of as-spun temperature sensor with other reported patch[1-4] or fiber sensors[5,6]. Yellow shadings indicate the patch biosensors and pink shadings indicate the fiber biosensors.

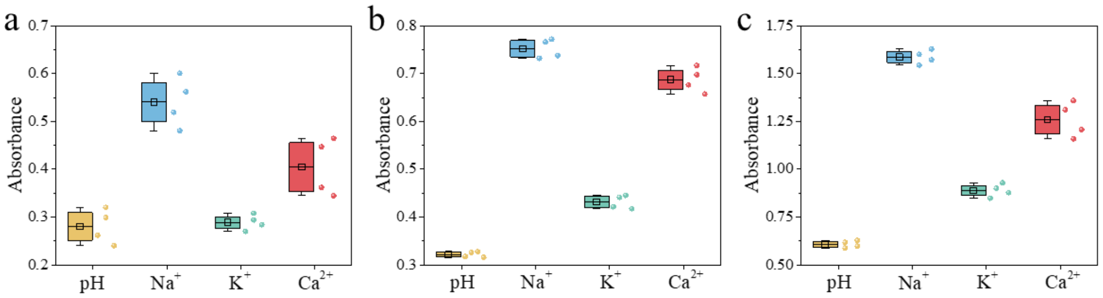

Supplementary Fig. 18 Box-and-whisker plot of mouse epithelial cells cultured in biocompatibility test. a-c CCK-8 experimental results within 24 h (a), 48 h (b) and (c) 72h (n = 4 independent experiments). Scale bar, 100 μm. Data are presented as mean values +/− SEM. The dashed lines indicate the upper and lower limits of the absorbance for the cells. The box ends represent the 25th and 75th percentiles. The horizontal line in each box represents the median. The upper and lower whiskers represent the maxima and minima, respectively, which refer to the range of non-outlier data values.

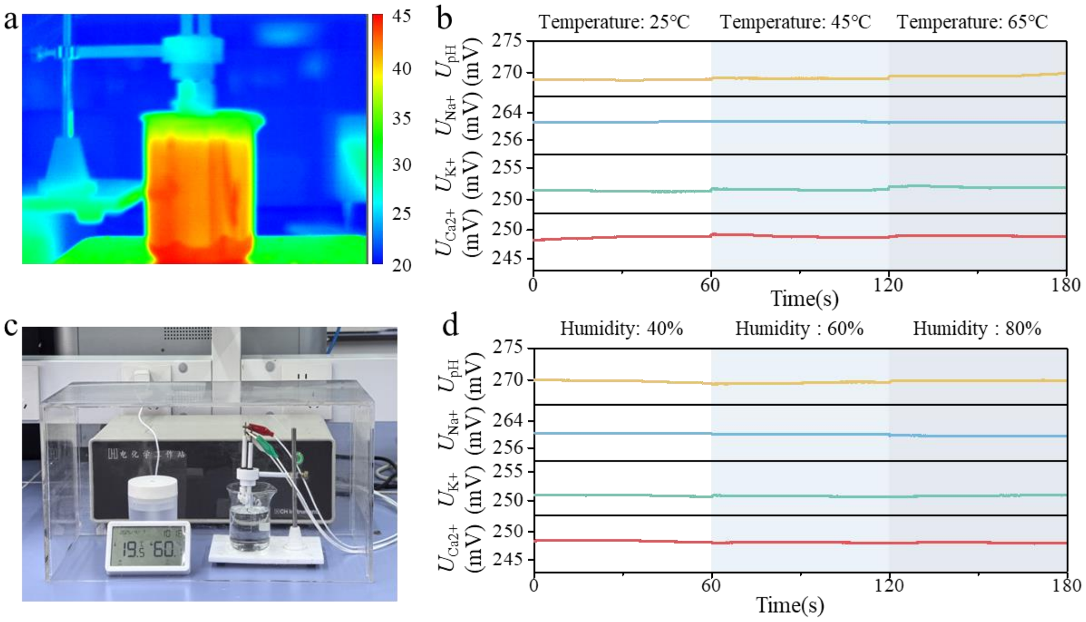

Supplementary Fig. 19 Stability of as-spun biosensors against environmental conditions. a, b Stability of the biosensors evaluated at different temperatures in a constant temperature heating table. c, d Stability of the biosensors evaluated under varying humidity conditions in a custom-built humidity box.

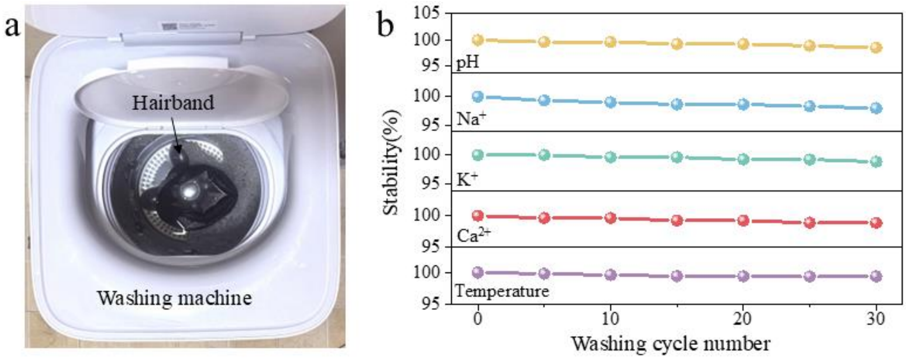

Supplementary Fig. 20 Stability of as-spun biosensors under regular washing. a Photograph of a multi-biosensing hairband being washed in a commercial washing machine. b Sensing stability of pH, Na^{+}, K^{+}, $Ca^{2+}$and temperature biosensors.

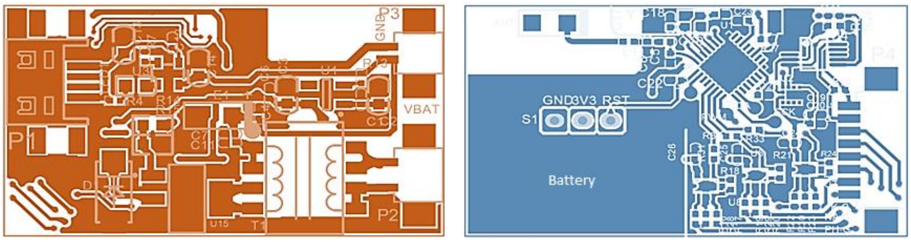

Supplementary Fig. 21 Schematic illustration and component list of the electronic system of circuit board.

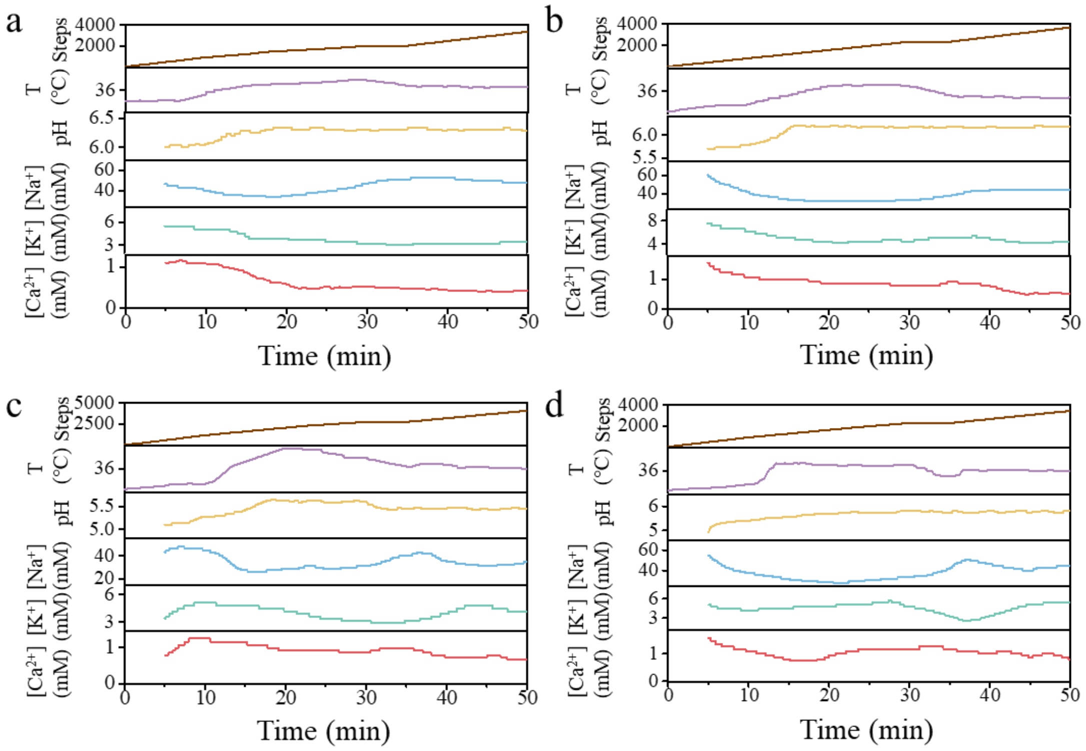

Supplementary Fig. 22 On-body multimodal monitoring for health monitoring during exercise. a-d Multimodal sensor responses in four healthy subjects using the multi-biosensing hairband during exercise.

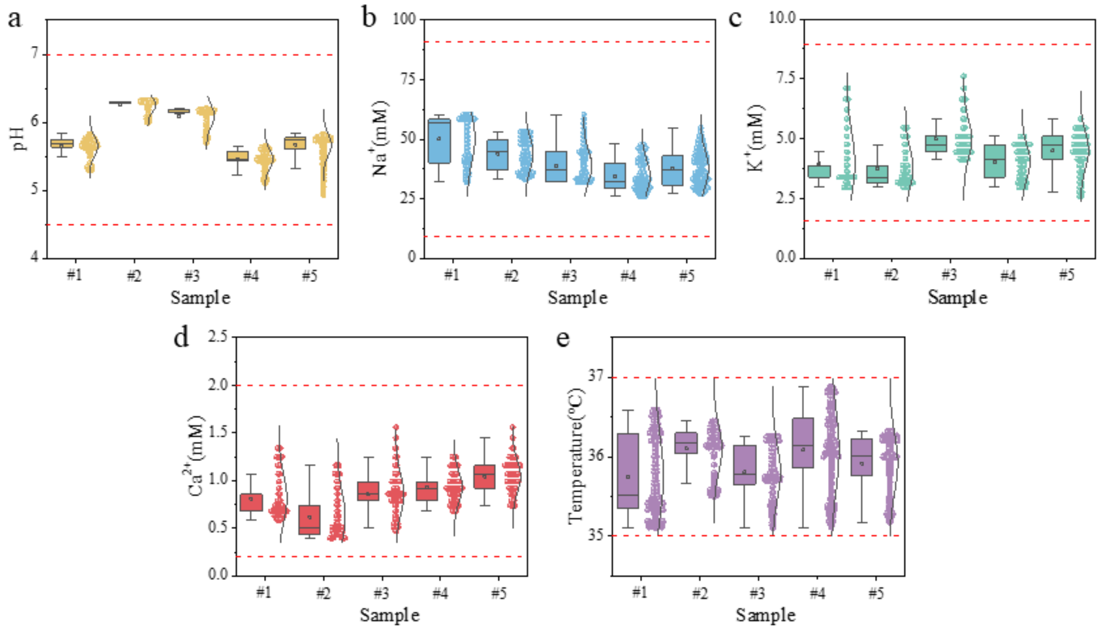

Supplementary Fig. 23 Distribution of physiological signals collected from five subjects during exercise. a-c Physiological signals of pH (a), Na^+ (b), K^+ (c), Ca^{2+} (d), and temperature (e) collected using the multi-biosensing hairband. The dashed lines indicate the upper and lower limits of the normal range for the biomarkers. The box ends represent the 25th and 75th percentiles. The horizontal line in each box represents the median. The upper and lower whiskers represent the maxima and minima, respectively, which refer to the range of non-outlier data values.

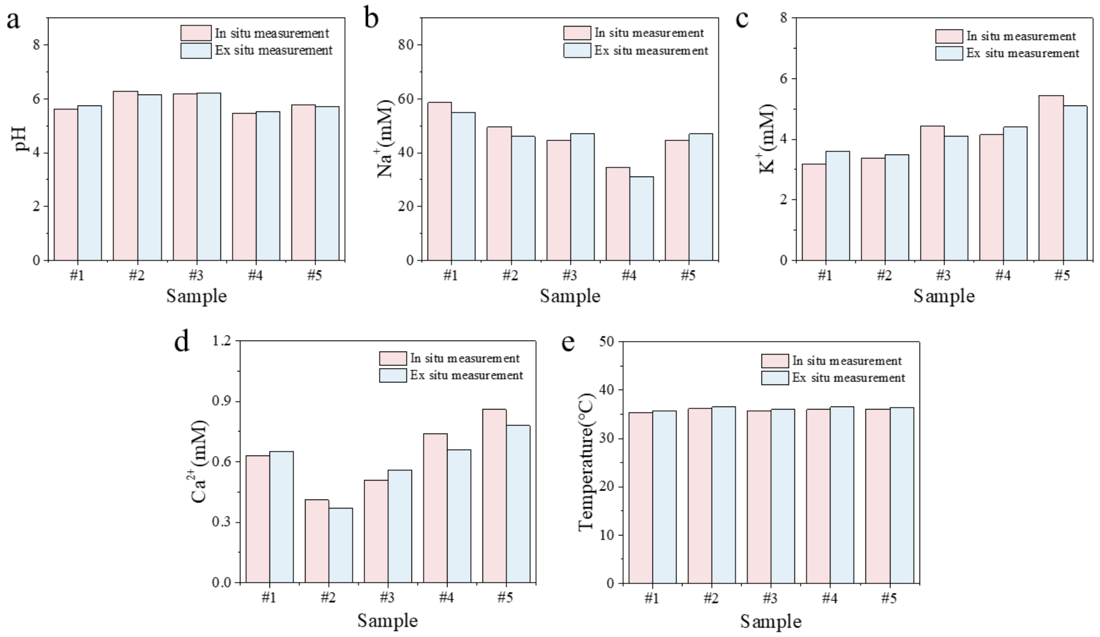

Supplementary Fig. 24 Ex situ measurements of physiological signals collected from five subjects during exercise. a-c Comparison of (a) pH, (b) Na^{+}, (c)K^{+}, (d)Ca^{2+} concentrations, and (e) temperature detected by the multi-biosensing hairband and standardized measurement.

Supplementary Table 1 Comparison of the temperature sensing performance

Substrate	Detection range (℃)	Sensitivity (% ℃$^{-1}$)	long-term stability (hours)	Ref.
PET	22-45	0.214	0.15	[1]
PI	28-90	0.113	-	[2]
Kapton	20-60	0.204	-	[3]
PI	30-100	0.05		[4]
Taffeta fabric	25-50	-1.04		[5]
Taffeta fabric	25-50	0.31	-	[6]
Silk/PLA/CNT yarn	22-45	0.268	24	This work

Supplementary Table 2 Comparison of the pH sensing performance

Substrate	Detection range	Sensitivity (mV pH^{-1})	long-term stability (hours)	Ref.
PVDF nanofiber	3.7-8.5	30.1	-	[7]
PVDF&PAN	3-8	29.3	-	[8]
CNT fiber	4-7	28	10	[9]
PDMS fiber	4-8	33.75	-	[10]
CNT fiber	4-7	38.3	1.39	[11]
Blend cloth	4-10	40±4	1	[12]
Silk/PLA/CNT yarn	3-7	39.52	24	This work

Supplementary Table 3 Comparison of the Na^+ sensing performance

Substrate	Detection range (mM)	Sensitivity (mV dec^{-1})	long-term stability (hours)	Ref.
CNT fiber	10-160	45.8	1.39	[11]
PVDF	10-160	31.3	-	[13]
PET	10-160	43.1	2	[14]
PDMS	5-80	46.66	0.56	[15]
PET	5-100	51.8	-	[16]
PI	10-160	48	-	[17]
Silk/PLA/CNT yarn	10-160	56.33	24	This work

Supplementary Table 4 Comparison of the K^+ sensing performance

Substrate	Detection range (mM)	Sensitivity (mV dec^{-1})	long-term stability (hours)	Ref.
CNT fiber	2-32	39	10	[9]
CNT fiber	2-32	35.9	1.39	[11]
PET	1.25-40	31.8	-	[16]
PI	0.625-40	30.42	3	[18]
TANi/GO fiber	2-32	26.9	-	[19]
PAN/PVP	0-32	34.7	1.67	[20]
Silk/PLA/CNT yarn	2-32	34.13	24	This work

Supplementary Table 5 Comparison of the Ca^{2+} sensing performance

Substrate	Detection range (mM)	Sensitivity (mV dec^{-1})	long-term stability (hours)	Ref.
CNT fiber	4-8	29.4	10	[9]
CNT fiber	0.5-2.53	52.3	1.39	[11]
PET	0.25-2	32.7	4	[21]
Circuit boards	10^{-4}−100	29.91	24	[22]
PVC	0.1-100	30	-	[23]
LIG	0.015-10	27.7	-	[24]
Silk/PLA/CNT yarn	0.5-2.53	30.61	24	This work

Supplementary Table 6 Personal information of five volunteers participating in the on-body sweat analysis using a multi-biosensing hairband.

Subject	Age	Gender	Height(cm)	Weight(kg)
#1	24	Male	178	75
#2	27	Female	158	50
#3	25	Male	176	67
#4	28	Female	165	62
#5	30	Male	176	78

Supplementary references

1. Ma, S. et al. Ultra-Sensitive and Stable Multiplexed Biosensors Array in Fully Printed and Integrated Platforms for Reliable Perspiration Analysis. *Adv. Mater.* **36**, 2311106 (2024).

2. Khalaf, A. M., Issa, H. H., RamíRez, J. L. & Mohamed, S. A. All Inkjet-Printed Temperature Sensors Based on PEDOT: PSS. *IEEE Access* **10**, 61094-61100 (2022).

3. Dankoco, M. D., Tesfay, G. Y., Benevent, E. & Bendahan, M. Temperature sensor realized by inkjet printing process on flexible substrate. *MSEB* **205**, 1-5 (2016).

4. Zhang, Y. et al. High-linearity graphene-based temperature sensor fabricated by laser writing. *J. Mater. Sci-Mater. El.* **35**, 109 (2024).

5. Kuzubasoglu, B. A., Sayar, E., Cochrane, C., Koncar, V. & Bahadir, S. K. Wearable temperature sensor for human body temperature detection. *J. Mater. Sci-Mater. El.* **32**, 4784-4797 (2021).

6. Kuzubasoglu, B. A., Sayar, E. & Bahadir, S. K. Inkjet-Printed CNT/PEDOT:PSS Temperature Sensor on a Textile Substrate for Wearable Intelligent Systems. *IEEE Sens. J.* **21**, 13090-13097 (2021).

7. Shi, S. et al. A Bionic Skin for Health Management: Excellent Breathability, In Situ Sensing, and Big Data Analysis. *Adv. Mater.* **36**, 2306435 (2024).

8. Liang, X. et al. Thermal Transfer Printed Flexible and Wearable Bionic Skin with Bilayer Nanofiber for Comfortable Multimodal Health Management. *Adv. Healthc. Mater.* **14**, 2403780 (2025).

9. Tian, H. et al. Hierarchical Fermat helix-structured electrochemical sensing fibers

enable sweat capture and multi-biomarker monitoring. *Mater. Horiz.* **10**, 5192-5201, (2023).

10. Mei, X., Yang, J., Liu, J. & Li, Y. Wearable, nanofiber-based microfluidic systems with integrated electrochemical and colorimetric sensing arrays for multiplex sweat analysis. *Chem. Eng. J.* **454**, 140248 (2023).

11. Wang, L. et al. Weaving Sensing Fibers into Electrochemical Fabric for Real-Time Health Monitoring. *Adv. Funct. Mater.* **28**, 1804456 (2018).

12. Salvo, P. et al. Temperature and pH sensors based on graphenic materials. *Biosens. Bioelectron.* **91**, 870-877 (2017).

13. Sun, Y. et al. Stretchable and Smart Wettable Sensing Patch with Guided Liquid Flow for Multiplexed in Situ Perspiration Analysis. *ACS Nano* **18**, 2335-2345 (2024).

14. Ji, W. et al. Large-scale fully printed "Lego Bricks" type wearable sweat sensor for physical activity monitoring. *NPJ Flex. Electron.* **7**, 53 (2023).

15. An, Z. et al. Body Heat Powered Wirelessly Wearable System for Real-time Physiological and Biochemical Monitoring. *Adv. Funct. Mater.* **33**, 2303361 (2023).

16. He, W. et al. Integrated textile sensor patch for real-time and multiplex sweat analysis. *Sci. Adv.* **5**, eaax0649 (2019).

17. Niu, J. et al. A Fully Elastic Wearable Electrochemical Sweat Detection System of Tree-Bionic Microfluidic Structure for Real-Time Monitoring. *Small* **20**, 2306769 (2024).

18. Gai, Y. et al. A Self-Powered Wearable Sensor for Continuous Wireless Sweat Monitoring. *Small Methods* **6**, 2200653 (2022).

19. Tong, X. et al. Multifunctional Fiber for Synchronous Bio-Sensing and Power Supply in Sweat Environment. *Adv. Funct. Mater.* **33**, 2301174 (2023).

20. Mo, L., Ma, X., Fan, L., Xin, J. H. & Yu, H. Weavable, large-scaled, rapid response, long-term stable electrochemical fabric sensor integrated into clothing for monitoring potassium ions in sweat. *Chem. Eng. J.* **454**, 140473 (2023).

21. Nyein, H. Y. Y. et al. A Wearable Electrochemical Platform for Noninvasive Simultaneous Monitoring of Ca^{2+} and pH. *ACS Nano* **10**, 7216-7224 (2016).

22. Cai, X. et al. Fully Integrated Multiplexed Wristwatch for Real-Time Monitoring of Electrolyte Ions in Sweat. *ACS Nano* **18**, 12808-12819 (2024).

23. Zareh, M. M., Mohamed, S. F. & Elsheikh, A. M. Polymeric Electrochemical Sensor for Calcium Based on DNA. *Polymers* **14**, 1896 (2022).

24. Soleimani, A. et al. Towards sustainable and humane dairy farming: A low-cost electrochemical sensor for on-site diagnosis of milk fever. *Biosens. Bioelectron.* 259, 116321 (2024).
