# Supplementary material for: Multi-biosensing hairband for emergency health assessment
Source: Nat Commun. 2025 Aug 5;16:7224. doi: 10.1038/s41467-025-62556-6 (PMC12325749; doi:10.1038/s41467-025-62556-6)
Supplement: Supplementary file 2 — Description of Additional Supplementary Information [file 41467_2025_62556_MOESM2_ESM.pdf]

Description of Additional Supplementary Files

File Name: Supplementary Video 1
Description: Continuous preparation of as-spun multi-biosensing yarns through the self-assembled coaxial wetspinning device.

File Name: Supplementary Video 2
Description: Viscoelastic comparison of carboxylated carbon nanotube, silk fibroin, polylactic acid, and SCP mixture.

File Name: Supplementary Video 3
Description: On body application of the multi-biosensing hairband during exercise.
