# Supplementary material for: Multi-biosensing hairband for emergency health assessment
Source: Nat Commun. 2025 Aug 5;16:7224. doi: 10.1038/s41467-025-62556-6 (PMC12325749; doi:10.1038/s41467-025-62556-6)
Supplement: Supplementary file 6 — Reporting Summary [file 41467_2025_62556_MOESM6_ESM.pdf]

nature portfolio

Corresponding author(s): Mingwei Tian
Last updated by author(s): Jul 4, 2025

Reporting Summary

Nature Portfolio wishes to improve the reproducibility of the work that we publish. This form provides structure for consistency and transparency in reporting. For further information on Nature Portfolio policies, see our Editorial Policies and the Editorial Policy Checklist.

Statistics

For all statistical analyses, confirm that the following items are present in the figure legend, table legend, main text, or Methods section.

n/a	Confirmed	
[ ]	[x]	The exact sample size (*n*) for each experimental group/condition, given as a discrete number and unit of measurement
[ ]	[x]	A statement on whether measurements were taken from distinct samples or whether the same sample was measured repeatedly
[x]	[ ]	The statistical test(s) used AND whether they are one- or two-sided<br>*Only common tests should be described solely by name; describe more complex techniques in the Methods section.*
[x]	[ ]	A description of all covariates tested
[x]	[ ]	A description of any assumptions or corrections, such as tests of normality and adjustment for multiple comparisons
[ ]	[x]	A full description of the statistical parameters including central tendency (e.g. means) or other basic estimates (e.g. regression coefficient) AND variation (e.g. standard deviation) or associated estimates of uncertainty (e.g. confidence intervals)
[ ]	[x]	For null hypothesis testing, the test statistic (e.g. *F*, *t*, *r*) with confidence intervals, effect sizes, degrees of freedom and *P* value noted<br>*Give P values as exact values whenever suitable.*
[x]	[ ]	For Bayesian analysis, information on the choice of priors and Markov chain Monte Carlo settings
[x]	[ ]	For hierarchical and complex designs, identification of the appropriate level for tests and full reporting of outcomes
[x]	[ ]	Estimates of effect sizes (e.g. Cohen's *d*, Pearson's *r*), indicating how they were calculated

Our web collection on statistics for biologists contains articles on many of the points above.

Software and code

Policy information about availability of computer code

Data collection	Sensing data were collected by an electrochemical workstation (CHI760e) and a parameter analyser (Keithley-2450). Characterization data were collected by Scanning Electron Microscope (SEM, ZEISSEVO18), Optical Contact Angle and 3D Shape Measurement system (DSA100), Rotational Rheometer (MCR702), Fourier transform infrared spectrometer (Nicolet IS10),Universal material testing machine (Instron 5300) and Specific surface area and aperture tester (ASAP-2460). On-body sweat data were collected by custom codes.
Data analysis	Data were analyzed through Origin 2015.

For manuscripts utilizing custom algorithms or software that are central to the research but not yet described in published literature, software must be made available to editors and reviewers. We strongly encourage code deposition in a community repository (e.g. GitHub). See the Nature Portfolio guidelines for submitting code & software for further information.

Data

Policy information about availability of data

All manuscripts must include a data availability statement. This statement should provide the following information, where applicable:

- Accession codes, unique identifiers, or web links for publicly available datasets
- A description of any restrictions on data availability
- For clinical datasets or third party data, please ensure that the statement adheres to our policy

All data supporting the findings of this study are available within the article and its supplementary files at https://doi.org/10.6084/m9.figshare.29465285. Any additional requests for information can be directed to, and will be fulfilled by, the corresponding authors. Source data are provided with this paper.

Custom code is available at https://github.com/Liming-dot/Multi-biosensing-hairband.

Research involving human participants, their data, or biological material

Policy information about studies with human participants or human data. See also policy information about sex, gender (identity/presentation), and sexual orientation and race, ethnicity and racism.

Reporting on sex and gender	Sex and gender information was only collected and shown as overall numbers for statistical information, which was irrelevant to the research findings. The sex and self-identified gender showed no discrepancies among the participants involved in this study.
Reporting on race, ethnicity, or other socially relevant groupings	The research findings were irrelevant to the race, ethnicity, or other socially relevant groupings of the participants.
Population characteristics	Five participants involved in this study were healthy adults (two females and three males, aging from 23-30 years old) with independent action abilities. Exclusion criteria included chronic diseases, mental disorder, skin allergies, medication, smoking, drinking, genetic metabolic disorders and other conditions that could interfere with the study.
Recruitment	Volunteers were recruited from Qingdao University and each participant gave informed written consent. The study was fully voluntary, and no compensation was given.
Ethics oversight	This study was approved by the Ethical Committee of the Affiliated Hospital of Qingdao University (QYFYWZLL30286).

Note that full information on the approval of the study protocol must also be provided in the manuscript.

Field-specific reporting

Please select the one below that is the best fit for your research. If you are not sure, read the appropriate sections before making your selection.

- [x] Life sciences
- [ ] Behavioural & social sciences
- [ ] Ecological, evolutionary & environmental sciences

For a reference copy of the document with all sections, see nature.com/documents/nr-reporting-summary-flat.pdf

Life sciences study design

All studies must disclose on these points even when the disclosure is negative.

Sample size	For on body evaluation of the multi-biosensing hairband, 5 healthy subjects(two females and three males, aging from 23-30 years old) were recruited. Sample sizes were chosen on the basis of literature standards for proof-of-concept experiments.
Data exclusions	No data were excluded from the analyses.
Replication	The study validated the sweat multimodal sensing, which was dependent on the body condition for each experiment session and was thus not replicated. However, for all the on-body evaluation of our wearable systems, the measurements were taken from five participants to demonstrate repeatability. All attempts at replication were successful when following the device-fabrication process described in this paper.
Randomization	The device was fabricated with same process and was tested in all participants under same conditions. Randomization was therefore not relevant to the study.
Blinding	Not relevant, because a blinding process wouldn't influence the sampling result.

Reporting for specific materials, systems and methods

We require information from authors about some types of materials, experimental systems and methods used in many studies. Here, indicate whether each material, system or method listed is relevant to your study. If you are not sure if a list item applies to your research, read the appropriate section before selecting a response.

Materials & experimental systems

n/a	Involved in the study	
x		Antibodies
	x	Eukaryotic cell lines
x		Palaeontology and archaeology
x		Animals and other organisms
x		Clinical data
x		Dual use research of concern
x		Plants

Methods

n/a	Involved in the study	
x		ChIP-seq
x		Flow cytometry
x		MRI-based neuroimaging

Eukaryotic cell lines

Policy information about cell lines and Sex and Gender in Research

Cell line source(s)	Normal murine fibroblast cells (L929, Procell)
Authentication	All cells were purchased from Procell and have been ethically sourced and authenticated thorough QC testing by Procell.
Mycoplasma contamination	We did not observe any mycoplasma contamination in our cell cultures.
Commonly misidentified lines (See ICLAC register)	N/A

Plants

Seed stocks	*Report on the source of all seed stocks or other plant material used. If applicable, state the seed stock centre and catalogue number. If plant specimens were collected from the field, describe the collection location, date and sampling procedures.*
Novel plant genotypes	*Describe the methods by which all novel plant genotypes were produced. This includes those generated by transgenic approaches, gene editing, chemical/radiation-based mutagenesis and hybridization. For transgenic lines, describe the transformation method, the number of independent lines analyzed and the generation upon which experiments were performed. For gene-edited lines, describe the editor used, the endogenous sequence targeted for editing, the targeting guide RNA sequence (if applicable) and how the editor was applied.*
Authentication	*Describe any authentication procedures for each seed stock used or novel genotype generated. Describe any experiments used to assess the effect of a mutation and, where applicable, how potential secondary effects (e.g. second site T-DNA insertions, mosiacism, off-target gene editing) were examined.*
